# Clinical Impact of Hormone Replacement Therapy on Atrial Fibrillation in Postmenopausal Women: A Nationwide Cohort Study

**DOI:** 10.3390/jcm10235497

**Published:** 2021-11-24

**Authors:** Jaehoon Lee, Yuntae Kim, Hyunji Park, Changsoo Kim, Sihyun Cho, Jongyoun Kim

**Affiliations:** 1 Department of Obstetrics and Gynecology, Division of Reproductive Endocrinology, Gangnam Severance Hospital, Yonsei University College of Medicine, Seoul 06273, Korea; jhlee126@yuhs.ac; 2 Department of Public Health, Yonsei University, Seoul 03722, Korea; yyyyt@yuhs.ac (Y.K.); localpark@yuhs.ac (H.P.); 3 Department of Preventive Medicine, Yonsei University College of Medicine, Seoul 03722, Korea; PREMAN@yuhs.ac; 4 Institute of Human Complexity and Systems Science, Yonsei University, Incheon 21983, Korea; 5 Cardiology, Heart Center, Gangnam Severance Hospital, Yonsei University College of Medicine, Seoul 06273, Korea

**Keywords:** atrial fibrillation, hormone replacement therapy, menopause, estrogen

## Abstract

Individuals with atrial fibrillation (AF), especially women, have an increased risk of stroke and death. Although hormone replacement therapy (HRT) is widely used in postmenopausal women, the association between HRT use and AF risk is unclear. We aimed to investigate the association between various types of HRT and AF. This was a population-based retrospective cohort study from The Korean National Health Insurance Service-National Sample Cohort (2004–2015). Participants were aged 45–60 years and were free from cardiovascular disease and AF at baseline. Overall, 13,452 (64.03%) women had never received HRT, 5671 (26.99%) had received HRT, and 1885 (8.98%) were currently receiving HRT. In multivariable analysis, the relative hazards for AF were significantly higher among current users (*p* < 0.001) and lower among past users (*p* = 0.069). Current users—except those using estradiol-only HRT—had significantly elevated AF risk. Among past users, only estradiol plus progestin HRT users had a reduced AF risk after adjusting for covariates (*p* = 0.027). Ongoing HRT posed an increased risk of AF. The degree of risk varied based on the specific type of estrogen and progestins co-administration. These findings indicate that, with respect to AF risk, oral estradiol-containing HRT is superior to HRT containing oral conjugated equine estrogen or tibolone.

## 1. Introduction

Atrial fibrillation (AF) is the most common sustained arrhythmia and a major public health problem. Individuals with AF have a five-fold increased risk of stroke, a four-fold increased risk of heart failure, and a nearly two-fold increased risk of dementia and death [1]. In particular, a higher risk of stroke and stroke-related mortality has been observed in women with AF than in men [2]. Although the pathophysiological mechanisms behind AF development are complex and not fully understood, there are several reports suggesting that female sex hormones modify AF risk in women [3]. 

Hormone replacement therapy (HRT) is used to control symptoms of menopause and prevent osteoporosis in menopausal women [4,5]. HRT was once used to prevent cardiovascular disease (CVD) [6,7,8,9]. However, after the release of Women’s Health Initiative (WHI) study results in the early 2000s, HRT was perceived as a factor that could increase CVD risk [10,11]. Successive extension studies of the WHI study have demonstrated that the risk of coronary artery disease (CAD) increases when HRT is started 10 years after the onset of menopause; starting HRT within 10 years of menopause onset lowers the risk (the so-called timing hypothesis) [12]. A recent Cochrane’s review also reported lower CAD and mortality rates among women who started HRT less than 10 years after menopause [13]. Although HRT is no longer administered solely to prevent CAD, CAD prevention is considered an added benefit of HRT.

Although the associations between HRT and CVD risk as well as CVD and AF risk have been studied rigorously, the risk between HRT and AF risk has not been studied sufficiently; this may be because many risk factors for AF are also contraindications for HRT. With underlying conditions such as old age, severe obesity, hypertension, diabetes, hypercholesterolemia, smoking, CAD, and valvular heart disease, which are risk factors for AF, HRT either cannot be administered or should be used with caution. To date, only three studies have examined these associations [14,15,16], which reported different AF risks according to the specific estrogen type and whether or not progestins were co-administered. In general, AF has a lower prevalence in women than in men [17,18,19], and longer lifetime exposure to endogenous sex hormones is significantly associated with a lower AF risk in women with natural menopause [20]. However, to date, it is difficult to draw a definitive conclusion regarding the association between female sex steroid hormones and AF risk. Moreover, although AF is less prevalent in women than in men, it is associated with higher risks of stroke and death in women [2,21,22,23]. Therefore, we aimed to investigate the association between various types of HRT and AF in a national cohort of Korean menopausal women.

## 2. Materials and Methods

### 2.1. Data Source and Ethical Considerations 

The South Korean government operates a mandatory nationwide insurance system that covers all forms of health services, including hospitalization, ambulatory care, and pharmaceutical services. The study population was recruited from the Korean National Health Insurance Service-National Sample Cohort (NHIS-NSC) database. Details of the NHIS-NSC database are described elsewhere [24]. Briefly, the NHIS-NSC database is a public database created by the NHIS that includes a sample (2.2%) of the South Korean population (approximately 1 million), that was extracted by systematic stratified random sampling with proportional allocation within 1476 strata constructed by age, group, sex, participant’s eligibility status, and income level to represent the entire population of South Korea. The disease information of the participants was classified according to the 10th revision of the International Classification of Disease codes by primary care physicians, as well as at the secondary and tertiary hospitals. The representativeness of the sample was examined by comparing the sample with the entire Korean population [25,26].

All identifiable personal data in the medical records were de-identified to comply with the Health Insurance Portability and Accountability Act privacy rule. The study protocol was approved by the Institutional Review Board of Gangnam Severance Hospital, Yonsei University College of Medicine, Korea (3-2020-0421). The institutional review board waived the requirement to obtain informed consent. This study was conducted according to the tenets of the Declaration of Helsinki.

### 2.2. Study Population

A total of 551,786 women enrolled in the NHIS-NSC who underwent medical examinations from 1 January 2004 to 31 December 2015, were included in this study. Women younger than 45 years or older than 60 years at baseline and those who started HRT after the age of 60 years were excluded (*n* = 446,555). Women with the following diagnoses of preexisting AF or risk factors for AF during the washout period were also excluded from the study: AF, *n* = 2373; acute myocardial infarction, *n* = 210; heart failure, *n* = 352; valvular heart disease, *n* = 87; cardiomyopathy, *n* = 10; CAD, *n* = 33; cerebrovascular accident, *n* = 815; chronic pulmonary disease, *n* = 11,965; severe liver disease, *n* = 359; severe diabetes mellitus, *n* = 1085; hemiplegia, *n* = 156; chronic renal disease, *n* = 169; malignancy, *n* = 2198. The washout period was defined as a period at least 1 year before the start of HRT for current and previous HRT users, and as 1 year after the start of observation for subjects who had never received HRT.

**Figure 1:**
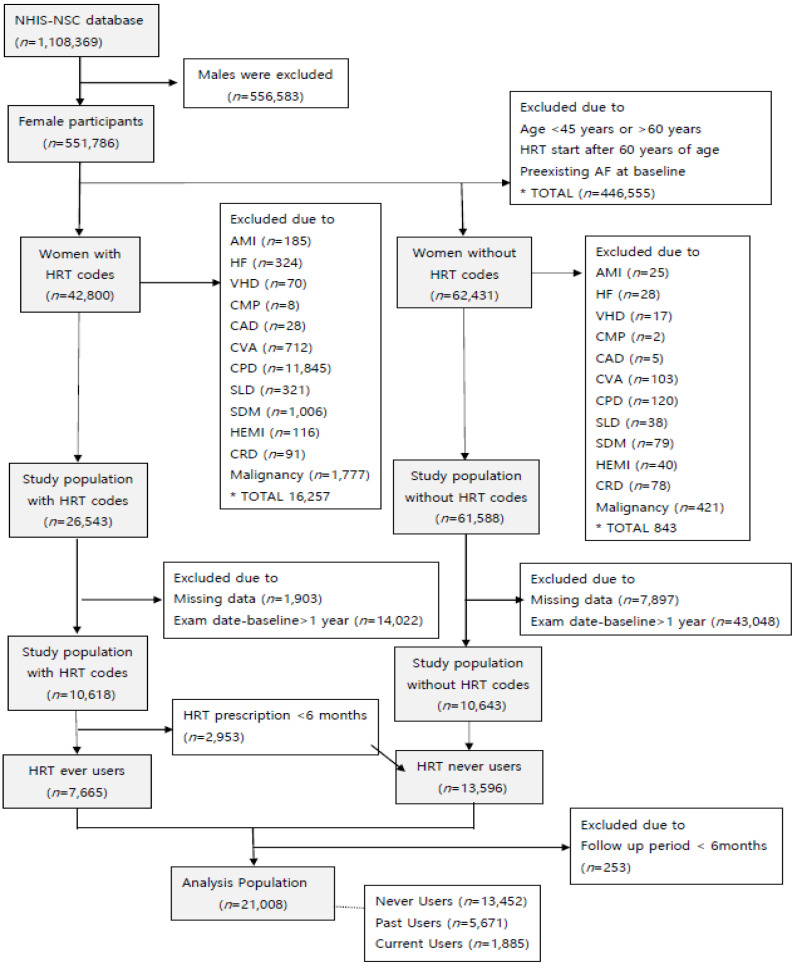
Flow chart of study participants.

If any of the covariates required for analysis were missing (*n* = 9800), or if there was a time interval of 1 year or more between the cohort entry date and the health examination date (*n* = 57,070), these women were excluded from the analysis. Finally, a total of 21,008 women between 45 and 60 years of age at baseline without preexisting AF or risk factors for AF, were included in this study (Figure 1). The International Classification of Disease codes (10th revision) for comorbidities and the outcome (AF) are presented in Table S1. 

### 2.3. Exposure to HRT

Women were divided into groups of HRT never users, past users, and current users. This is a classical approach to examine the effects of HRT use on study outcomes [27,28]. Women who had been prescribed HRT for more than 6 months and used more than two HRT prescriptions within the past 6 months were defined as current users. Women who used fewer than two HRT prescriptions within the past 6 months were defined as previous users. Women with no HRT prescriptions or those who had been prescribed HRT for less than 6 months during the study period were defined as never users. Types of HRT were classified as estrogen-only HRT, estrogen plus progestin HRT, and tibolone. Estrogen-only HRT included oral conjugated equine estrogen (CEE) and oral estradiol (E2). Estrogen plus progestin HRT included oral CEE plus progestin and oral E2 plus progestin. Generally, estrogen-only HRT is restricted to women who have undergone hysterectomy, and estrogen plus progestin HRT and tibolone are prescribed to women with a uterus. When two or more HRT types were administered, the regimen used for the longest period was used for classifying the patient into a group. Since the number of women who used transdermal E2 was too small, they were excluded from the analyses. The prescription codes for HRT are presented in Table S1.

### 2.4. Statistical Analysis 

The characteristics of the groups defined by HRT use were compared using the chi-square test and one-way analysis of variance. We calculated the incidence rate per 1000 person-years and 95% confidence interval (CI) to compare the AF incidence of HRT users to that of non-users. Multiple comparisons were adjusted using Bonferroni’s method. We obtained multivariable adjusted hazard ratios (HR)s and 95% CIs for new-onset AF incidence between the groups defined by HRT use by using the Cox regression model with time-dependent covariates to reduce the immortal time bias. All statistical analyses were performed using SAS Enterprise Guide^®^ (SAS Institute Inc., Cary, NC, USA). All tests were two-tailed, with *p* < 0.05 considered significant.

## 3. Results

### 3.1. Baseline Characteristics

Patient characteristics are summarized in Table 1. In the overall population, 13,452 (64.03%) women had never used HRT, 5671 (26.99%) women had used HRT previously, and 1885 (8.98%) women were currently using HRT. At baseline, the mean age was 51.10 ± 4.08 years for HRT never users, which was significantly higher than that for the HRT current users (50.18 ± 3.51 years). The mean follow-up periods were 11.42 ± 3.55 years for HRT never users, 8.94 ± 3.84 years for HRT past users, and 6.02 ± 3.97 years for HRT current users. Among HRT users, the mean duration of HRT use was 2.55 ± 2.39 years for current users and 0.90 ± 1.30 years for past users.

### 3.2. AF and HRT Use

Table 2 shows the incidence rates of new-onset AF among HRT never users, past users, and current users. Among 381 participants with new-onset AF, 279 were never users, 68 were past users, and 34 were current users. Raw AF incident rates were 1.82 per 1000 person-years for never users, 1.34 per 1000 person-years for past users, and 2.56 per 1000 person-years for current users.

When adjusted for age, HRT current users had a significantly higher AF incidence rate than never users (3.00 vs. 1.82 per 1000 person-years). Consistent results were obtained (HR: 2.17, 95% CI: 1.50–3.13; *p* < 0.001) after adjusting for baseline characteristics (Model 1). Because heart disease is a major risk factor for AF development, we further adjusted for newly diagnosed acute myocardial infarction, heart failure, valvular heart disease, and cardiomyopathy during the study period (model 2). Despite adjusting for these newly diagnosed heart diseases, a similar trend was observed among current users (HR: 2.24; 95% CI: 1.55–3.23; *p* < 0.001). However, in past users, a lower AF incidence rate was observed than that in the never users, when corrected for age (1.34 vs. 1.82 per 1000 person-years). This trend was consistent in Model 1 (HR: 078; 95% CI: 0.59–1.01; *p* = 0.063) and in Model 2 (HR: 0.78; 95% CI: 0.60–1.02; *p* = 0.069).

### 3.3. AF in HRT Current Users

Table 3 shows the incidence rate and multivariable adjusted HRs of new-onset AF according to the type of HRT for current users. The crude incidence of AF in estrogen plus progestin HRT users and tibolone users was significantly higher than that in never users, whereas it was not for estrogen-only HRT users. These results were consistent even after adjusting for baseline characteristics and new-onset cardiac diseases (Table 3, Model 2; estrogen-only HRT users: HR: 2.04, 95% CI: 0.84–4.97, *p* = 0.115; estrogen plus progestin HRT users: HR: 2.30, 95% CI: 1.30–4.06, *p* = 0.004; tibolone users: HR: 2.23, 95% CI: 1.34–3.73, *p* = 0.002).

However, the AF risk differed based on the type of estrogen used and whether or not progestogenic drugs were co-administered. For current users, an increased AF risk was observed for all HRT users except for E2-only HRT users (Table 4, Model 2; HR: 1.45, 95% CI: 0.46–4.53, *p* = 0.524). These results were only minimally affected by adjustments for patient characteristics and incident cardiovascular events (Table 4, Model 2; CEE-only HRT users: HR: 5.35, 95% CI: 1.32–21.66, *p* = 0.019; E2 plus progestin HRT users: HR: 2.30, 95% CI: 1.30–4.07, *p* = 0.004; tibolone users: HR: 2.23, 95% CI: 1.34–3.73, *p* = 0.002). 

### 3.4. AF in HRT Past Users

For past users, reduced AF risk was observed in estrogen plus progestin HRT users compared to that in never users (Table 5, Model 2; HR: 0.60; 95% CI: 0.38–0.94; *p* = 0.026). Among estrogen plus progestin HRT users, E2 plus progestin HRT users had a decreased risk of AF compared to HRT never users after adjusting for covariates (Table 6, Model 2; HR: 0.57; 95% CI: 0.35–0.94; *p* = 0.027), whereas no significant difference was observed in CEE plus progestin HRT users (Table 6, Model 2; HR: 0.75, 95% CI: 0.24–2.35, *p* = 0.626). 

The risk of AF was not significantly different between past users of estrogen-only HRT and HRT never users during the study period (Table 5, Model 2; HR: 0.81; 95% CI: 0.48–1.37; *p* = 0.431). Estrogen-only HRT neither increased nor decreased AF risk regardless of whether CEE or E2 was the estrogen administered (Table 6, Model 2; CEE-only HRT users: HR: 0.92, 95% CI: 0.51–1.64, *p* = 0.768; E2-only HRT users: HR: 0.55, 95% CI: 0.12–1.74, *p* = 0.311). Tibolone also did not increase the risk of AF (HR: 0.94; 95% CI: 0.65–1.35; *p* = 0.730).

## 4. Discussion

Our results showed that current HRT use was associated with an increased AF risk for women between 45 and 60 years of age without any underlying disease. Past HRT use was not associated with an increased AF risk. The degree of AF risk varied based on the specific estrogen type and whether or not progestins were co-administered. Among HRT current users, an increased AF risk was observed for all HRT types, except E2 alone. Among HRT past users, an increased AF risk was not observed for any HRT type, and a reduced AF risk was observed for women who had used E2 plus progestin. Since estrogen-only HRT can be administered only in women who have undergone hysterectomy, considering the results of this study comprehensively, HRT containing E2 is more beneficial than CEE or tibolone in terms of AF risk for both current and past users.

The main finding of this study partially agrees with findings of previous studies. Perez et al., who performed an extension study of the WHI study, reported that women randomized to estrogen-only HRT were at an increased HF risk (HR: 1.17; 95% CI: 1.00–1.36; *p* = 0.045) compared to women randomized to placebo, while estrogen plus progestin HRT users were not (HR: 1.07; 95% CI: 0.91–1.25; *p* = 0.44) [14]. Since CEE was the only estrogen used in the WHI studies, these results are consistent with findings of this study, wherein the AF increase was observed in women currently receiving CEE-only HRT. Similarly, Wong et al. continued to observe participants of the Women’s Health Study and found that estrogen monotherapy was associated with an increased AF risk (HR: 1.22; 95% CI: 1.02–1.45; *p* = 0.028), whereas estrogen and progestin co-treatment were not (HR 1.04; 95% CI 0.86–1.26; *p* = 0.69) [15]. Among previous studies, only that by Tsai et al. assessed AF risk based on the specific estrogen type [16]. In the study by Tsai et al., a higher AF risk was observed for CEE users than for E2 users (HR: 1.96; 95% CI: 1.03–3.73; *p* = 0.042). 

It is difficult to directly compare the results of this study with those of previous studies for several reasons. First, the difference in results may have been caused by the different subgroup classifications of the study population. None of the aforementioned studies distinguished between HRT current users and past users, which is a classical approach to examining the effects of HRT use on study outcomes. Wong et al. classified their study population into HRT current users and past users; however, this subgroup classification was not reflected in their outcome analyses [15]. 

Second, the previous studies included a large number of older women, which also may have caused the differences in results. The baseline age of the subjects was 63.3 years in the study by Perez et al. [14], 53.1 years in the study by Wong et al. [15], and 54.9 years in the study by Tsai et al. [16]. In contrast, the baseline age was 50.9 years in this study. After the publication of the WHI study results, which were used by Perez et al. [14], it was pointed out that the WHI study included a large number of older women; therefore, the CVD incidence increased rapidly after HRT administration. It is unknown whether AF is significantly affected by the age when HRT is started; however, CAD, an AF risk factor, is significantly affected by the age when HRT is started [29,30,31]. Because the leading menopause societies recommend starting HRT before age 60 years [4,5], women who started receiving HRT after age 60 years were excluded from this study to represent real-world clinical practice more accurately.

Third, the general practice patterns of the United States and South Korea are different. CEE is preferred in the United States, whereas estradiol is more often administered in Korea. The study by Tsai et al. [16]. did not evaluate how past and current HRT use influenced AF occurrence differently and did not consider whether progestin was co-administered. However, theirs was the only study that assessed AF risk based on the specific estrogen type. Previous clinical data have shown that CEE results in longer-lasting metabolites and may cause inflammation, whereas transdermal E2 has a short half-life and is not pro-inflammatory [32].

Considering the fact that AF is a significant risk factor for stroke [32,33], especially in women [2,21,22,23], an increase in AF incidence following HRT may be a clue to understanding the association between HRT and stroke, which is controversial [13,16,33,34,35,36]. It is not fully understood why starting HRT within 10 years of menopause reduces CAD risk, but not the stroke incidence [12,13,30,31,35,36,37]. The Nurse’s Health study reported an increased relative risk of ischemic stroke for current users of both estrogen-only HRT (relative risk: 1.43; 95% CI: 1.17–1.74) and estrogen plus progestin HRT (relative risk: 1.53; 95% CI: 1.21–1.95) [36]. In their study, a comparison between HRT initiation near menopause onset and HRT initiation at 10 or more years after menopause showed no significant difference in the results. The higher AF incidence rate observed in this study may be a causal factor behind higher stroke incidence reported among the current HRT users. The association among HRT, AF, and stroke warrants further evaluation.

The mechanisms behind the associations observed in this study remain unclear. Estrogen can affect cardiac ion channel dynamics [38], and postmenopausal women have different repolarization kinetics and atrial electrophysiological properties than their premenopausal counterparts [39,40,41,42]. In this study, we postulate that HRT has a promoting effect on AF during administration, and AF risk returns to baseline after discontinuing HRT. Although the past users would have been at a similar risk for new-onset AF to that of current users while receiving HRT, the mean duration of HRT use was significantly shorter among past users (327.37 ± 475.79 days) than among current users (930.56 ± 871.19 days). In contrast, the mean follow-up period was significantly longer for past users (3263.79 ± 1401.50 days) than for current users (2196.15 ± 1449.33 days). After discontinuing HRT, the AF risk may have decreased owing to the general cardio-protective effect of HRT.

In this study, while the AF risk did not change in women currently receiving E2-only HRT, an increase in AF risk was observed in CEE users, estrogen plus progestin HRT users, and tibolone users. CEE consists of a complex mixture of at least 10 estrogens. Progestins have various extents of glucocorticoid, mineralocorticoid, and androgenic properties. Finally, tibolone is known to convert into various metabolites with properties similar to estrogen, progestin, and androgen, and shows affinity for both glucocorticoid receptors and mineralocorticoid receptors. Considering these points, the findings of this study suggest that the AF risk may manifest through non-genomic mechanisms of estrogen or other steroid actions mediated by receptors of progesterones, glucocorticoids, or mineralocorticoids, rather than effects through estrogen receptors.

To increase the accuracy of diagnosis in this study, certain underlying diseases were analyzed using diagnostic codes, prescription codes, and medical procedure codes together. An important part of this analysis was accurately ascertaining the primary outcome (AF). A previous study using the NHIS-NSC indicated that the AF diagnosis was validated, with a positive predictive value of 94.1% [43]. However, this study has several limitations. Since our database was significantly large, we could not confirm therapy compliance among women receiving HRT. In addition, differences in the AF incidence according to the HRT administration route or the specific progestin type were not considered because of the lack of statistical power since there were only a few subjects. Finally, the number of AF cases was too small in certain subgroups because we performed analysis according to the estrogen type and progestin. Because the risk estimates were based on only two AF cases in current CEE users, caution must be exercised in result interpretation.

## 5. Conclusions

In this national cohort study of women without preexisting AF and risk factors for AF, current HRT use was associated with an increased risk of AF, while past HRT use was not. Notably, an increased AF risk was observed for all HRT types, except E2-only HRT. These findings suggest the possibility that AF risk may manifest via the effects of other steroid actions rather than effects via estrogen receptors. Therefore, for women with risk factors for AF, an HRT regimen containing E2 may be recommended rather than CEE or tibolone. Since the AF risk was not sustained and even decreased after stopping HRT, there seems to be no reason to refrain from prescribing HRT in women with indications for HRT.

## Figures and Tables

**Table 1:** Baseline characteristics.

	Total (*n* = 21,008)	Never Users (*n* = 13,452)	Past Users (*n* = 5671)	Current Users (*n* = 1885)	*p* Value
Mean follow-up period, days	3747.59 (1478.30)	4168.95 (1295.09)	3263.79 (1401.50)	2196.15 (1449.33)	<0.001
Age, years	50.99 (3.93)	51.10 (4.08)	51 (3.64)	50.18 (3.51)	<0.001
BMI, kg/m^2^	23.74 (3)	23.98 (3.08)	23.51 (2.82)	22.76 (2.64)	<0.001
Height, cm	155.69 (5.18)	155.46 (5.22)	155.93 (5.1)	156.57 (4.96)	<0.001
Weight, kg	57.56 (7.75)	57.96 (7.97)	57.18 (7.37)	55.80 (6.93)	<0.001
SBP	122 (17.08)	123.02 (17.57)	120.79 (16.46)	118.39 (14.50)	<0.001
DBP	76.35 (11.25)	76.89 (11.48)	75.73 (10.95)	74.38 (10.07)	<0.001
Total cholesterol	202.48 (42.22)	202.86 (45.05)	202.54 (37.01)	199.62 (35.35)	0.008
HTN, *n* (%)	7193 (34.24)	4881 (36.28)	1868 (32.94)	444 (23.55)	<0.001
Diabetes, *n* (%)	2073 (9.87)	1497 (11.13)	474 (8.36)	102 (5.41)	<0.001
HCL, *n* (%)	2999 (14.28)	1966 (14.61)	814 (14.35)	219 (11.62)	0.004
HRT, *n* (%)					<0.001
E only	1222 (5.82)	0 (0)	984 (17.35)	238 (12.63)	
E + P	3279 (15.61)	0 (0)	2440 (43.03)	841 (44.62)	
Tibolone	3049 (14.51)	0 (0)	2247 (39.62)	806 (42.76)	
Never	13,458 (64.06)	13,458 (100)	0 (0)	0 (0)	
Mean duration of HRT use, days	447.85 (653.68)	-	327.37 (475.79)	930.56 (871.19)	<0.001
Smoking, *n* (%)					<0.001
Current	583 (2.78)	312 (2.32)	184 (3.24)	87 (4.62)	
Former	227 (1.08)	107 (0.80)	81 (1.43)	39 (2.07)	
Never	20,198 (96.14)	13,033 (96.89)	5406 (95.33)	1759 (93.32)	
Alcohol consumption, *n* (%)					<0.001
Rarely/never	18,413 (87.65)	12,178 (90.53)	4811 (75.52)	1424 (75.54)	
1–2 drinks/week	2016 (9.6)	974 (7.24)	679 (19.32)	363 (19.26)	
3–4 drinks/week	383 (1.82)	185 (1.38)	122 (4.04)	76 (4.03)	
>5 drinks/week	196 (0.93)	115 (0.85)	59 (1.12)	22 (1.17)	
Exercise, *n* (%)					<0.001
Rarely/never	11,347 (54.01)	8027 (59.67)	2679 (47.24)	641 (34.01)	
1–2 times/week	5268 (25.08)	2974 (22.11)	1621 (28.58)	673 (35.70)	
3–4 times/week	2687 (12.79)	1379 (10.25)	906 (15.98)	402 (21.33)	
5–6 times/week	640 (3.05)	340 (2.53)	194 (3.42)	106 (5.62)	
7 times/week	1066 (5.07)	732 (5.44)	271 (4.78)	63 (3.34)	

Abbreviations: BMI, body mass index; SBP, systolic blood pressure; DBP, diastolic blood pressure; HTN, hypertension; HRT, hormone replacement therapy; E, estrogen; P, progestin; HCL, hypercholesterolemia.

**Table 2:** AF risk according to HRT use.

	HRT Use			
	Total	Never	Past	Current
Number of AF cases	381	279	68	34
Person-years	215,697.10	153,645.81	50,709.50	11,341.79
AF incidence rate * (95% CI)	1.77 (1.60–1.95)	1.82 (1.61–2.04)	1.34 (1.06–1.70)	3.00 (2.14–4.19)
Age-adjusted AF incidence rate * (95% CI)	1.77 (1.76–1.78)	1.82 (1.81–1.83)	1.34 (1.32–1.35)	3.00 (2.52–2.62)
Age-adjusted HR (95% CI)		Reference	0.78 (0.60–1.01)	2.01 (1.40–2.88)
			*p* = 0.063	*p* < 0.001
Model 1 †		Reference	0.78 (0.59–1.01)	2.17 (1.50–3.13)
			*p* = 0.063	*p* < 0.001
Model 2 ‡		Reference	0.78 (0.60–1.02)	2.24 (1.55–3.23)
			*p* = 0.069	*p* < 0.001

Abbreviations: AF, atrial fibrillation; HRT, hormone replacement therapy; CI, confidence interval; HR, hazard ratio. * Per 1000 patient-years. † Model 1: adjusted for age, body mass index, height, hypertension, diabetes, hypercholesterolemia, smoking status, alcohol consumption, and exercise. ‡ Model 2: additionally adjusted for cardiovascular events occurring prior to AF onset.

**Table 3:** AF risk for HRT current users according to HRT type.

		HRT Type (Current Users)			
	Total (*n* = 15,337)	None (*n* = 13,452)	E Only (*n* = 238)	E + P (*n* = 841)	Tibolone (*n* = 806)
Number of AF cases	313	279	5	13	16
Person-years	164,987.60	153,645.81	1570.46	4673.92	5097.41
AF incidence rate * (95% CI)	1.90 (1.70–2.12)	1.82 (1.61–2.04)	3.18 (1.33–7.64)	2.78 (1.62–4.79)	3.14 (1.92–5.12)
Age-adjusted AF incidence rate * (95% CI)	1.90 (1.89–1.91)	1.82 (1.81–1.83)	3.20 (3.05–3.34)	2.81 (2.73–2.89)	3.12 (3.04–3.20)
Age-adjusted HR (95% CI)		Reference	2.05 (0.85–4.98)	1.96 (1.12–3.45)	2.02 (1.21–3.35)
			*p* = 0.11	*p* = 0.02	*p* = 0.007
Model 1 †		Reference	1.95 (0.80–4.74)	2.25 (1.28–3.97)	2.15 (1.29–3.59)
			*p* = 0.141	*p* = 0.005	*p* = 0.004
Model 2 ‡		Reference	2.04 (0.84–4.97)	2.30 (1.30–4.06)	2.23 (1.34–3.73)
			*p* = 0.115	*p* = 0.004	*p* = 0.002

Abbreviations: HRT, hormone replacement therapy; E, estrogen; P, progestin; AF, atrial fibrillation; CI, confidence interval; HR, hazard ratio. * Per 1000 patient-years. † Model 1: adjusted for age, body mass index, height, hypertension, diabetes, hypercholesterolemia, smoking status, alcohol consumption, and exercise. ‡ Model 2: additionally adjusted for cardiovascular events occurring prior to AF onset.

**Table 4:** AF risk for HRT current users according to the estrogen type and progestin co-treatment.

		HRT Regimen (Current Users)					
	Total (*n* = 15,337)	None (*n* = 13,452)	CEE (*n* = 48)	E2 (*n* = 190)	E2 + P (*n* = 841)	CEE + P (*n* = 0)	Tibolone (*n* = 806)
Number of AF cases	313	279	2	3	13	0	16
Person-years	164,987.60	153,645.81	293.34	1277.12	4673.92	-	5097.41
AF incidence rate * (95% CI)	1.90 (1.70–2.12)	1.82 (1.61–2.04)	6.82 (1.71–27.13)	2.35 (0.76–7.27)	2.78 (1.62–4.79)		3.14 (1.92–5.12)
Age-adjusted AF incidence rate * (95% CI)	1.90 (1.89–1.91)	1.82 (1.81–1.83)	7.91 (7.33–8.48)	2.35 (2.21–2.49)	2.81 (2.73–2.89)		3.12 (3.04–3.20)
Age-adjusted HR (95% CI)		Reference	4.48 (1.11–18.04)	1.51 (0.48–4.71)	1.96 (1.12–3.45)	-	2.02 (1.21–3.35)
			*p* = 0.035	*p* = 0.479	*p* = 0.019		*p* = 0.007
Model 1 †		Reference	4.95 (1.22–20.05)	1.39 (0.44–4.34)	2.25 (1.28–4.00)		2.15 (1.29–3.60)
			*p* = 0.025	*p* = 0.573	*p* = 0.005		*p* = 0.003
Model 2 ‡		Reference	5.35 (1.32–21.66)	1.45 (0.46–4.53)	2.30 (1.30–4.07)		2.23 (1.34–3.73)
			*p* = 0.019	*p* = 0.524	*p* = 0.004		*p* = 0.002

Abbreviations: HRT, hormone replacement therapy; CEE, conjugated equine estrogen; E2, estradiol; P, progestin; AF, atrial fibrillation; CI, confidence interval; HR, hazard ratio. * Per 1000 patient-years. † Model 1: adjusted for age, body mass index, height, hypertension, diabetes, hypercholesterolemia, smoking status, alcohol consumption, and exercise. ‡ Model 2: additionally adjusted for cardiovascular events occurring prior to AF onset.

**Table 5:** AF risk for HRT past users according to HRT type.

		HRT Type (Past Users)			
	Total (*n* = 19,123)	None (*n* = 13,452)	E Only (*n* = 984)	E + P (*n* = 2440)	Tibolone (*n* = 2247)
Number of AF cases	347	279	15	20	33
Person-years	204,355.31	153,645.81	9819.02	20,901.44	19,989.04
AF incidence rate * (95% CI)	1.90 (1.53–1.89)	1.82 (1.61–2.04)	1.53 (0.92–2.53)	0.28 (0.62–1.48)	1.65 (1.17–2.32)
Age-adjusted AF incidence rate * (95% CI)	1.70 (1.89–1.91)	1.82 (1.81–1.83)	1.51 (1.47–1.55)	0.96 (0.93–0.98)	1.65 (1.62–1.68)
Age-adjusted HR (95% CI)		Reference	0.85 (0.51–1.43)	0.59 (0.37–0.93)	0.93 (0.65–1.34)
			*p* = 0.543	*p* = 0.022	*p* = 0.693
Model 1 †		Reference	0.80 (0.48–1.35)	0.60 (0.38–0.94)	0.93 (0.65–1.34)
			*p* = 0.411	*p* = 0.026	*p* = 0.705
Model 2 ‡		Reference	0.81 (0.48–1.37)	0.60 (0.38–0.94)	0.94 (0.65–1.35)
			0.85 (0.51–1.43)	0.59 (0.37–0.93)	0.93 (0.65–1.34)

Abbreviations: HRT, hormone replacement therapy; E, estrogen; P, progestin; AF, atrial fibrillation; CI, confidence interval; HR, hazard ratio. * Per 1000 patient-years. † Model 1: adjusted for age, body mass index, height, hypertension, diabetes, hypercholesterolemia, smoking status, alcohol consumption, and exercise. ‡ Model 2: additionally adjusted for cardiovascular events occurring prior to AF onset.

**Table 6:** AF risk for HRT past users according to the estrogen type and progestin co-treatment.

	HRT Regimen (Past Users)						
	Total (*n* = 19,123)	None (*n* = 13,452)	CEE (*n* = 541)	E2 (*n* = 443)	E2 + P (*n* = 2286)	CEE + P (*n* = 154)	Tibolone (*n* = 2247)
Number of AF cases	347	279	12	3	17	3	33
Person-years	204,355.31	153,645.81	6273.93	3545.09	18,883.21	2018.24	19,989.04
AF incidence rate * (95% CI)	1.70 (1.53–1.89)	1.82 (1.61–2.04)	1.91 (1.09–3.37)	0.85 (0.27–2.62)	0.90 (0.56–1.45)	1.49 (0.48–4.60)	1.65 (1.17–2.32)
Age-adjusted AF incidence rate * (95% CI)	1.70 (1.89–1.91)	1.82 (1.81–1.83)	1.89 (1.84–1.95)	0.85 (0.80–0.90)	0.90 (0.88–0.92)	1.48 (1.40–1.57)	1.65 (1.62–1.68)
Age-adjusted HR (95% CI)		Reference	1.00 (0.56–1.78)	0.54 (0.17–1.67)	0.57 (0.35–0.92)	0.76 (0.24–2.36)	0.93 (0.65–1.33)
			*p* = 0.994	*p* = 0.282	*p* = 0.023	*p* = 0.632	*p* = 0.691
Model 1 †		Reference	0.91 (0.51–1.62)	0.55 (0.18–1.71)	0.58 (0.35–0.94)	0.74 (0.24–2.30)	0.93 (0.65–1.34)
			*p* = 0.747	*p* = 0.301	*p* = 0.028	*p* = 0.599	*p* = 0.702
Model 2 ‡		Reference	0.92 (0.51–1.64)	0.55 (0.12–1.74)	0.57 (0.35–0.94)	0.75 (0.24–2.35)	0.94 (0.65–1.35)
			*p* = 0.768	*p* = 0.311	*p* = 0.027	*p* = 0.626	*p* = 0.730

Abbreviations: HRT, hormone replacement therapy; CEE, conjugated equine estrogen; E2, estradiol; P, progestin; AF, atrial fibrillation; CI, confidence interval; HR, hazard ratio. * Per 1000 patient-years. † Model 1: adjusted for age, body mass index, height, hypertension, diabetes, hypercholesterolemia, smoking status, alcohol consumption, and exercise. ‡ Model 2: additionally adjusted for cardiovascular events occurring prior to AF onset.

## Data Availability

All data generated or analyzed during this study are included in this published article or in the data repositories listed in the References. The present study utilized the sample cohort database, which is third-party data, not owned by the authors. The sample cohort database is available upon approval for data sharing from the health insurance corporation. For the purposes of policy and academic research, a fee is paid to obtain the data from the NHIS website (https://nhiss.nhis.or.kr). Access date: 30 July 2021.

## Supplementary Materials

The following are available online at https://www.mdpi.com/article/10.3390/jcm10235497/s1, Table S1: Ascertainment of disease and medication.
