# Supplementary material for: Clinical Impact of Hormone Replacement Therapy on Atrial Fibrillation in Postmenopausal Women: A Nationwide Cohort Study
Source: J Clin Med. 2021 Nov 24;10(23):5497. doi: 10.3390/jcm10235497 (PMC8658255; doi:10.3390/jcm10235497)
Supplement: Supplementary file 1 [file jcm-10-05497-s001.zip › jcm-1394526-supplementary.pdf]

Table S1. Ascertainment of disease and medication.

Outcome		Definition by ICD-10
Atrial fibrillation		I48
**Comorbidities**		**Definition by ICD-10**
Acute myocardial infarction		I21, I25.2
Heart failure		I50
Valvular heart disease		I05,I34,I06,I35
Cardiomyopathy		I42.0, I42.1, I42.2
Cerebrovascular disease		I60-64, I69
Chronic pulmonary disease		J42-J47, J60-67, J70.1, J70.3
Severe liver disease		K70.3, K71.7, K72.1, K72.9, K74.3-6, I85, I86.4, I98.2
Severe diabetes mellitus		E10.2, E10.3, E10.4, E10.5, E10.7, E11.2, E11.3, E11.4, E11.5, E11.7, E12.2, E12.3, E12.4, E12.5, E12.7, E13.2, E13.3, E13.4, E13.5, E13.7, E14.2, E14.3, E14.4, E14.5, E14.7
Hemiplegia		G04.1, G11.4, G80.1, G81, G82, G83.0-4, G83.9
Chronic renal disease		N18, Z94.0, Z49.1, Z49.2, Z99.2, T861
Malignancy		C00-97
Coronary artery disease		Women who were diagnosed with coronary artery disease and even received interventional or surgical treatments.
	Diagnosis (ICD-10)	I20, I22, I23, I24, I25.1, I25.3, I25.4, I25.5, I25.6, I25.7, I25.8, I25.9
	Interventional or surgical treatments (procedure code)	M6551,M6552,M6561,M6562,M6563,M6564,M6571,M6572,M6601,M6602,O1641,O1642,O1647,OA641,OA642,OA647
**Medication**		**Definition by prescription code**
Estrogen-only HRT		Women who have been prescribed estrogen, but not progestin.
Estrogen	CEE	155401ATB, 155402ATB, 155404ATB
	E2	155001ATB, 155002ATB, 154901ATB, 154903ATB
	transdermal E2	154931CCM, 154604CPC
Progestin	Cyproterone	139401ATB
	Dienogest	615701ATB
	Dydrogesterone	150501ATB
	LNG-IUS	183401CIM
	Megestrol acetate	189301ASS, 189301ATB, 189302ATB, 189303ASS, 189304ASS, 189330ASS, 189331ASS, 189335ASS, 202901ATB
	MPA	188903ATB, 188904ATB, 188905ATB, 188906ATB
	MPA depot injection	188902BIJ
	Micronized progesterone	195001ACS

Estrogen+progestin HRT	Women who have been prescribed both estrogen and progestin.
CEE+MPA	298100ATB,465500ATB, 453500ATB, 297200ATB, 297300ATB
E2+Cyproterone acetate	398400ATB
E2+Drosprienone	490400ATB
E2+dydrogesterone	297400ATB, 433700ATB, 433800ATB
E2+norethisterone acetate	297100ATB, 297000ATB, 507600ATB, 298200ATB
E2+MPA	433900ATB, 434000ATB, 434100ATB, 297600ATB

Abbreviation: ICD-10, International Classification of Diseases, 10th edition; HRT, hormone replacement therapy; CEE, conjugated equine estrogens; E2, estradiol; LNG-IUS, levonorgestrel-releasing intrauterine system; MPA, medroxyprogesterone acetate: P, progestin.
